# Research on Mechanisms and Controlling Methods of Macro Defects in TC4 Alloy Fabricated by Wire Additive Manufacturing

**DOI:** 10.3390/ma11071104

**Published:** 2018-06-28

**Authors:** Lei Ji, Jiping Lu, Shuiyuan Tang, Qianru Wu, Jiachen Wang, Shuyuan Ma, Hongli Fan, Changmeng Liu

**Affiliations:** 1 School of Mechanical Engineering, Beijing Institute of Technology, Beijing 100081, China; jl12199@163.com (L.J.); jipinglu@bit.edu.cn (J.L.); qrwu@foxmail.com (Q.W.); bitmc@bit.edu.cn (S.M.); fanhongli2000@sina.com (H.F.); liuchangmeng@bit.edu.cn (C.L.); 2 Institute of Advanced Structure Technology, Beijing Institute of Technology, Beijing 100081, China; jcwang666@163.com

**Keywords:** wire feeding additive manufacturing, wire lateral feeding, macro defects, side spatters

## Abstract

Wire feeding additive manufacturing (WFAM) has broad application prospects because of its advantages of low cost and high efficiency. However, with the mode of lateral wire feeding, including wire and laser additive manufacturing, gas tungsten arc additive manufacturing etc., it is easy to generate macro defects on the surface of the components because of the anisotropy of melted wire, which limits the promotion and application of WFAM. In this work, gas tungsten arc additive manufacturing with lateral wire feeding is proposed to investigate the mechanisms of macro defects. The results illustrate that the defect forms mainly include side spatters, collapse, poor flatness, and unmelted wire. It was found that the heat input, layer thickness, tool path, and wire curvature can have an impact on the macro defects. Side spatters are the most serious defects, mainly because the droplets cannot be transferred to the center of the molten pool in the lateral wire feeding mode. This research indicates that the macro defects can be controlled by optimizing the process parameters. Finally, block parts without macro defects were fabricated, which is meaningful for the further application of WFAM.

## 1. Introduction

Wire feeding additive manufacturing is a very promising technology [1]. Compared with powder additive manufacturing, all the materials are sent to the welding pool in a lateral wire feeding mode. Consequently, the utilization of materials is extremely high, which can reduce wastage of materials and improve fabricating efficiency [2,3,4]. Moreover, materials in the form of wire are cheaper than powders. Therefore, WFAM is particularly suitable for manufacturing large scale structures or other complicated structures in aerospace [5].

According to the types of heat source, WFAM can be divided into wire and laser additive manufacturing (WLAM) [6,7], electron beam freeform fabrication (EBF) [8,9], gas tungsten arc welding (GTAW)-based wire arc additive manufacturing (WAAM) [10,11,12] and pulse arc welding (PAW)-based wire arc additive manufacturing [5,13].

**Figure 1:**
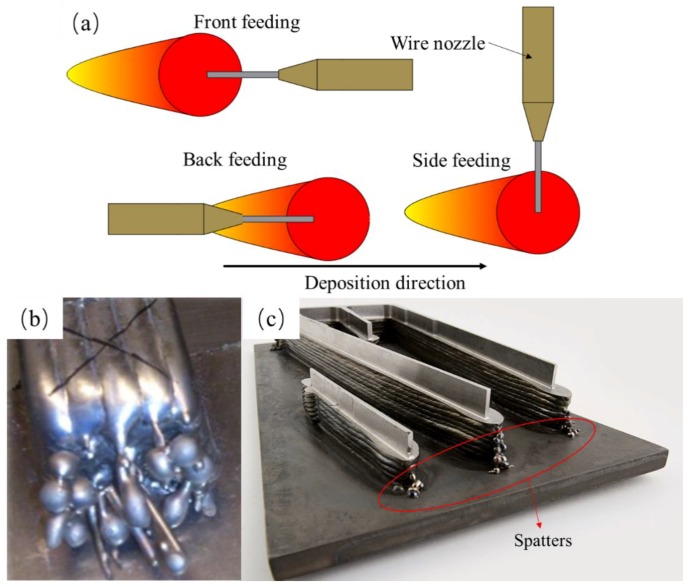
Schematic of the (**a**) lateral wire feeding in different feeding direction [3], “the ugly” samples (**b**) referred by P. Hagqvist, and component [15] (**c**) fabricated by Norsk Titanium [16].

However, in these WFAM methods, owing to the anisotropy of the lateral wire feeding method, the wire is not melted uniformly [14], as the wire is sent into the welding pool from the side of the arc zone (as Figure 1a [3]). In the lateral wire feeding mode, the uneven melting of wire could lead to macro defects of the components, such as spatters and unmelted wire (Figure 1b), which are common and inevitable phenomena in WFAM. Hagqvist found that the process is hard to control during laser metal deposition, creating parts with spatters and unmelted phenomena that he called “the ugly” [15]. Norsk Titanium is the pioneering supplier of aerospace-grade, additive manufactured, structural titanium components [16]. Spatters and rough surfaces also exist in its components, as indicated in Figure 1c [17]. Much follow-up processing is needed even if the component is a thin-walled part. Yongzhe Li [18] devised a layers-overlapping strategy to improve the accuracy of components fabricated by wire arc additive manufacturing. Temperature field control [19] and passive-vision [20] were used to improve the accuracy of the sample. Meanwhile, if the wire is heated under improper parameters, the wire cannot be heated uniformly, and non-uniform melting will lead to changes in the size of the welding pool, which may also lead to other defects. Much research has been conducted on the microstructure and mechanical properties of components fabricated using WFAM [7,8,10,21], but little research on the macro defects of WFAM has been reported. However, these kinds of macro defects always restrict the development of WFAM. Reducing or eliminating those defects through adjusting process parameters would significantly promote the development of WFAM. 

This work aimed to find the mechanisms and controlling methods of the macro defects fabricated by wire arc additive manufacturing, based on previous research on high-efficiency fabricating processes of GTAW-based wire arc additive manufacturing for TC4 titanium alloy. Four sets of experiments were set up to investigate the effects of heat input, layer thickness, tool path, and wire curvature. The effect of these parameters on the defects was analyzed systematically. The formation mechanisms of side spatters, collapse, unmelted wire and poor flatness were investigated, and controlling methods based on the process are presented.

## 2. Experimental Details

**Figure 2:**
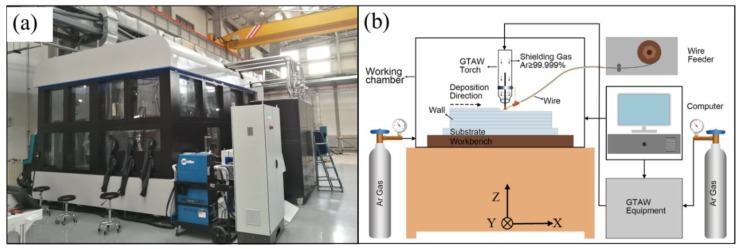
(**a**) Overview of wire and arc additive manufacturing system and (**b**) schematic of the equipment. GTAW = gas tungsten arc welding.

The equipment used in this research was developed independently by Beijing Institute of Technology (Figure 2a), a schematic is shown in Figure 2b [11]. The developed wire arc additive manufacturing system mainly consists of a wire feeder, a computer, a working chamber, and a GTAW machine. Normally, the parts were fabricated under protective conditions with an argon atmosphere of 99.99%.

**Figure 3:**
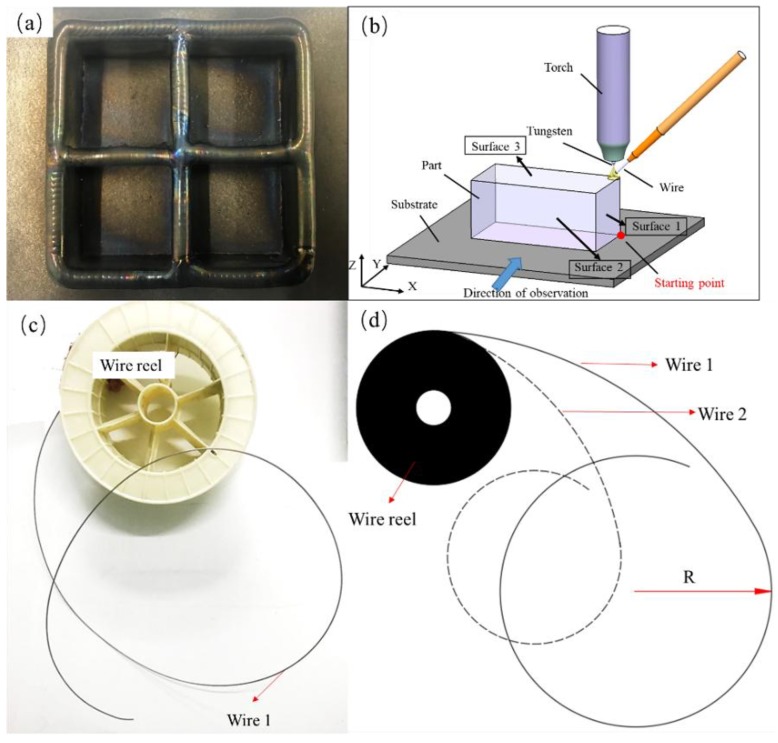
(**a**) Sample fabricated previously [4]; (**b**) schematic of the relationship between the part, base plate, and the welding torch; (**c**) wire reel and wire with curvature and (**d**) schematic of the wire with different curvature.

**Figure 4:**
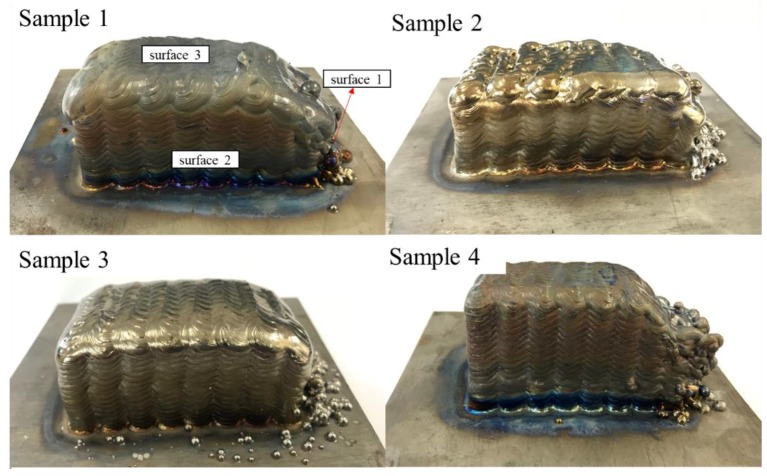

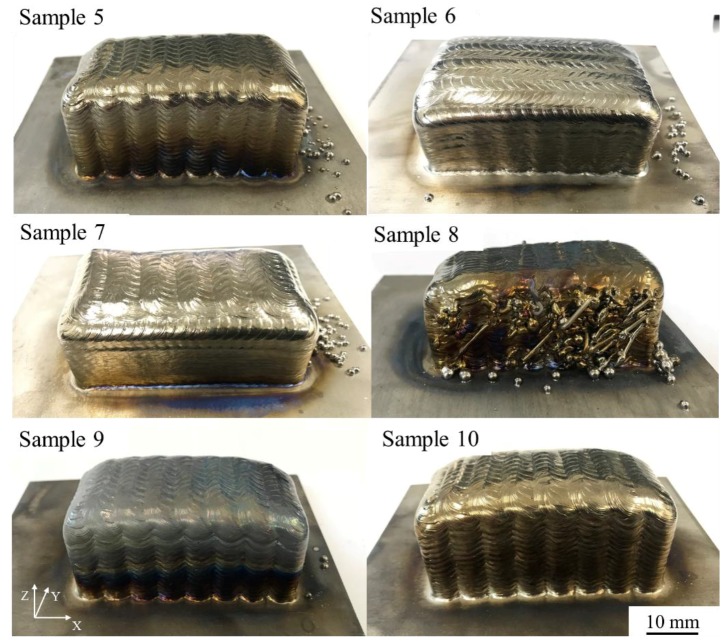
Macro images of the ten samples fabricated by using wire and arc additive manufacturing.

Based on previous research, in this research we obtained a relatively mature weld bead process and a thin-walled part molding process. The parts in this research were fabricated based on these processes. A large amount of research on the wire arc additive manufacturing process with 1.6-mm wire has been conducted. Figure 3a [4] shows a typical part with thin walls fabricated previously. Although numerous parameters may result in macro defects, according to actual fabricating experience, four parameters had a great influence on the macro defects and were chosen to investigate the mechanisms and controlling methods. As given in Table 1, the variables of heat input, layer thickness, tool path, and wire curvature were used to design four sets of experiments. The wire curvature is defined as the bending degree of the wire in its unrestrained state (Figure 3c). In mathematics, the curvature of the circle can be regarded as the reciprocal of the radius approximatively. So the radii of wires with different curvature were measured to calculate the curvature (Figure 3d). Block parts with a size of about 80 × 40 × 32 mm were fabricated by wire arc additive manufacturing in this work, and the macro defects existing in them were studied. To describe the research expediently, the relationship between the part, substrate, and the welding torch is shown in Figure 3b. The welding torch with the wire attached at a fixed angle (45°) moves under a computer numerical control (CNC) system. The wire feeding method is lateral paraxial feeding. In the following description, surfaces 1, 2, and 3 are named for the convenience of description. The parameters used in this research are listed in Table 1. Finally, ten samples (1–10) of four experiment groups are shown in Figure 4. Especially, the surfaces of samples 1, 4, and 9 look dark gray because of poor argon protection. This poor argon protection was unintentional and was the result of the chamber being opened too early, causing the parts to oxidize during processing. However, each layer was fabricated in an argon atmosphere without oxidation, so only the surface of the parts was oxidized. Because this research is aimed at the existence of defects on the macro level, this factor can be ignored in the research.

## 3. Results and Discussions

### 3.1. Effect of Heat Input on Macro Defects

#### 3.1.1. The Form of Defects

**Figure 5:**
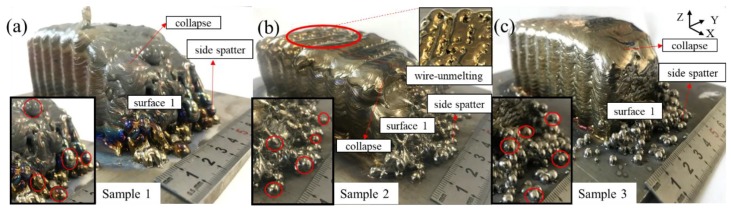
Macro images of three samples with different heat inputs: (**a**) 261 J/mm; (**b**) 173 J/mm; and (**c**) 217 J/mm.

Figure 5 shows the defects existing in three samples with different heat inputs. It can be seen that the defects are mainly located on surface 1. For sample 1, spatter of a large size occurs on the first few layers of surface 1, and there is a serious metal flowing phenomenon that causes the collapse of surface 1. For sample 2, spatter occurs a bit later, and its size appears to be small compared to that of sample 1. The degree of collapse has also been significantly reduced. However, the wire is not melted sufficiently on surface 3, as the enlarged view of Figure 5b shows. In sample 3, the spatter occurs in the last few layers and the number of spatters decreases significantly, thereby reducing the degree of collapse of surface 1.

#### 3.1.2. Macro Defect Mechanisms with Different Heat Inputs

In Figure 5, there are three types of defects, i.e., side collapse, side spatters, and unmelted wire, respectively. To analyze these phenomena, the following equations [10,22,23] are used to calculate the value of the heat input by regarding the difference in heat input as the difference in current.

(1) $$Hi\frac{J}{mm}=\eta I_{av}\frac{V_{av}}{TS}$$

(2) $$I_{av}\left(A\right)=\frac{I_{p}t_{p}+I_{b}t_{b}}{t_{p}+t_{b}}$$

where *Hi* is the heat input per unit length, η is the arc efficiency (assumed to be 0.83) for tungsten inert gas (TIG) welding [10], *V_av_* is the average of the instantaneous arc voltage, *I_av_* is the average current for pulse current TIG welding, *V_av_* is the average of the instantaneous arc voltage, *TS* is the travel speed, *t_p_* is the peak time, *t_b_* is the base current duration, *I_p_* is the peak current, and *I_b_* is the base current.

**Figure 6:**
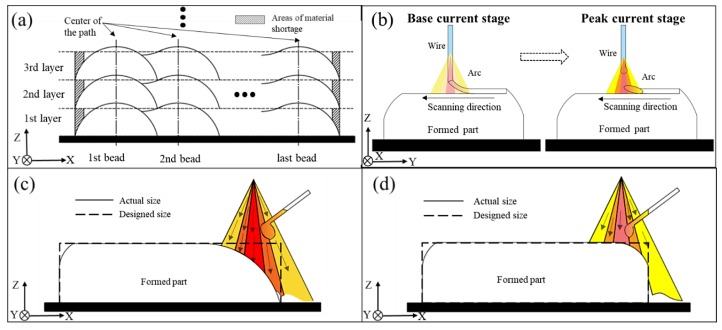
Schematics of (**a**) multi-layer overlapping; (**b**) the wire melted under a low heat input; (**c**) deposition process with excessive heat input and (**d**) deposition process with a low heat input.

As a consequence, the heat input to samples 1, 2, and 3 are 261, 173, and 217 J/mm, respectively. Combined with these values, the defect mechanisms are discussed as follows:

1. Side collapse is mainly caused by an overlapping process and excessive heat input. When fabricating the multi-layer, multi-bead blocks, the paths used are coincident in every layer. Therefore, the blocks’ edge is lower than the blocks’ main body. As Figure 6a shows, Li [24] demonstrated that material shortage areas are generated at the edges of the blocks when two layers overlap. With the increase of the number of layers, material shortage areas can accumulate, and finally the blocks begin to collapse. Moreover, excessive heat input can cause serious collapse because of the metal flow along the wall. As Figure 6c,d shows, with decreasing heat input, the degree of collapse can be significantly reduced and the actual size of the part becomes closer to the designed size.
2. Side spatters are mainly caused by the lateral wire feeding mode and excessive heat input. The wire is always inserted from one side, which can easily generate side spatters. When the process parameters are not matched with each other, the metal droplets can easily be splashed under the arc force in a fastigiated direction.
  As Figure 6c shows, when the heat input is 261 J/mm, the wire melts away from the center of the arc. Because the arc temperature is fairly high, the wire can easily be melted and the droplets generated at the front of the wire are larger and larger. Once at the pulsed current stage, the droplet falls under the arc force in a fastigiated direction, causing the droplet to be blown to the outside. The part material is lessened as well, especially at the edge of the block, so the droplets cannot fall into the center of the path with the lower surface. Therefore, the droplets are splashed as side spatters. When the heat input is decreased to 216 and 173 J/mm, the wire is more likely to be sent to the center of the arc and the size of the droplet is diminished, as Figure 6d shows. Once at the pulsed current stage, the droplet can be blown to the path more accurately.
  As shown in Figure 5, five droplets with a clear appearance are selected for each sample and the average size is calculated. The spatter sizes of samples 1, 2, and 3 in Figure 5 are 6.39, 4.21, and 4.25 mm. That is, even if the heat input is reduced, spatter still exists because of the special side feeding mode, but the size of the spatters and their number can be reduced efficiently.
3. The wire cannot be fully melted when the heat input is too small. As Figure 6b shows, a large amount of wire material is not melted at the base current, with the wire traveling against the formed surface. Once at the peak current, the wire is directly fused to the formed surface, causing the wire not to be fully melted and resulting in poor bonding between the contiguous weld beads. When the heat input is insufficient, the parts are not well formed and the wire feeding system becomes unstable during manufacturing and the stability of part forming is reduced, owing to the front of the wire touching the surface.

### 3.2. Effect of Layer Thickness on Macro Defects

#### 3.2.1. The Form of Defects

**Figure 7:**
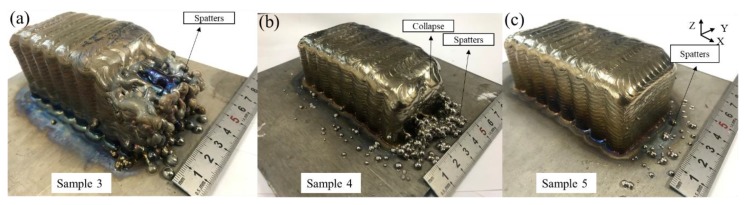
Macro images of samples 3, 4, and 5 with different layer thicknesses: (**a**) 1.1 mm; (**b**) 1.05 mm; and (**c**) 1.05/1.1 mm.

Figure 7 shows the defects existing in three samples with different layer thicknesses. It can be clearly seen that the defects are mainly located on surface 1 in the form of side spatters. The side spatters of sample 3 occur early and the number of spatters is great. Sample 4 has fewer and smaller spatters than sample 3. Surface 1 in sample 5 has a small number of spatters with smaller size, and the outline of the sample is clear as well.

#### 3.2.2. Macro Defect Mechanisms with Different Layer Thicknesses

**Figure 8:**
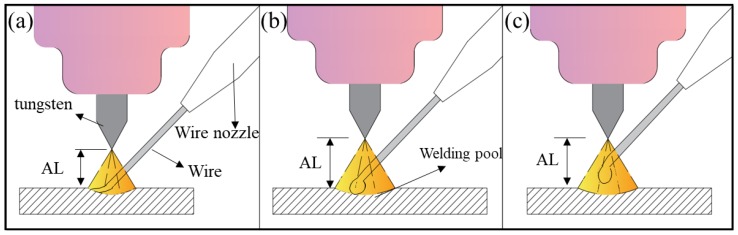
Schematic of (**a**) no-droplet mode; (**b**) tangent-droplet mode and (**c**) no-contact mode.

The type of transfer mode depends on the arc length (AL), which is the distance from the front of the tungsten to the fabricated surface (Figure 8). The definition of the layer thickness is the height lifted by the machine of every layer in the fabricating process. Therefore, in wire arc additive manufacturing, the setting of the layer thickness has an accumulative effect on the AL. The value of the layer thickness is regarded with the height of the weld bead. There is no doubt that the height of the weld bead is determined by the heat input and wire feed speed, but these other process parameters are the same in each sample. Therefore, the following equation [25] is used to calculate the height of the weld bead, *h*, in these process parameters:

(3) $$h=\frac{3\pi v_{w}d_{w}^{2}}{8wv_{t}}$$

where *w* is the width of the weld bead, $v_{w}$ is the wire feed speed, *d_w_* is the wire diameter, $v_{t}$ is the welding speed. The layer thickness is then calculated as 1.12 mm. Given that the condition of heat dissipation is different from that for an ideal weld bead, the preset layer thicknesses are 1.05, 1.05/1.1, and 1.1 mm, which lead to three different modes (Figure 8).

1. Figure 8a shows the no-droplet mode with small AL. The wire is fed into the bottom of the molten pool during the fabricating process, which means that the wire is in contact with the bottom of the molten pool during the feeding as in continuous molding. Opderbecke and Guiheux mentioned the existence of a liquid bridge, which contrasts with the no-contact mode. In this mode, the metal in the molten pool is mainly controlled by surface tension and gravity and remains stable, so each weld bead has a relatively flat appearance [26]. Compared with other modes, this mode can lower the height of the weld bead, reflected as collapse when the block part is fabricated.
2. As the AL increases, as shown in the Figure 8b, the tangent-droplet mode dominates. Here the wire does not touch the bottom of the molten pool during the feeding process. The front of the wire is melted, then a droplet is formed that adheres to the front of the wire owing to its own surface tension. The droplet becomes larger and larger until it is in contact with the molten pool. Owing to its own gravity and the arc force under the peak current, the droplet falls off the front of the wire and falls onto the formed surface to complete the metal transition from the wire smoothly.
3. Figure 8c shows the no-contact mode with large AL. The wire is further away from the bottom of the molten pool. Similarly, a droplet forms at the base current and sticks to the front of the wire. Necking phenomenon occurs as a result of gravity, and the droplet is stretched but cannot be in contact with the molten pool. Such droplets are blown to the formed surface by the arc force until the pulsed current works. In this mode, magnetic bias blowing occurs, and this easily leads to the splash of the droplets. At the same time, droplets in this mode are relatively close to the tungsten, and may pollute the tungsten and reduce the service life and fabricating stability.

As sample 3 in Figure 7 shows, a layer thickness of 1.1 mm is selected, although the metal transfer mode of the first few layers is the tangent-droplet mode (Figure 8b). As the number of layers increases, the cumulative error becomes larger, leading to the AL becoming larger in the later layers. The transfer mode changes to the no-contact mode (Figure 8c), resulting in the occurrence of spatters. When the layer thickness is reduced to 1.05 mm, as sample 4 in Figure 7 shows, the initial layers are still via the tangent-droplet mode (Figure 8b), and the layers are still well formed at a height of 1.05 mm. Similarly, as the number of layers increases, the cumulative error makes the AL become too small, and the metal transfer mode is changed to the no-droplet mode (Figure 8a). Although this can reduce the number of droplets, the wire is always at the bottom of the molten pool. As a result, unfused wire may exist on the surface or inside of the part. Moreover, the wire always touches the fabricated surface in the process, which can produce a certain reaction force on the wire feeding system.

After taking the above effects into account, a variable layer thickness is adopted. When the first layers are fabricated at the initial stage, the substrate temperature is low, so a 1.05 mm layer thickness is set to ensure the tangent-droplet (Figure 8b) mode, which can ensure that the droplet falls on the predefined path and lays a good foundation of appearance for the later layers. To ensure that the AL is maintained at a certain distance in tangency-droplet mode (Figure 8b) in the remaining layers, a layer thickness of 1.1 mm is used for the rest of the layers. As sample 5 in Figure 7 shows, after changing the layer thickness, it can be clearly seen that the spatters of surface 2 are pretty rare and the collapse phenomenon is also improved a lot. Obviously, the adjustment of the layer thickness plays a significant role in controlling the existence of side spatters.

### 3.3. Effect of Tool Path on Macro Defects

#### 3.3.1. The Form of Defects

**Figure 9:**
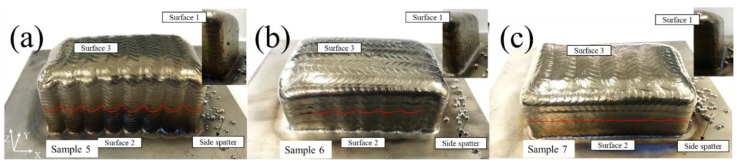
Macro images of samples 5, 6, and 7 with different tool paths: (**a**) Z-shaped path; (**b**) vertical path; and (**c**) contour-filling path.

Figure 9 shows the defects existing in three samples with different tool paths. It can be clearly seen that the defects are mainly in the form of poor flatness, where samples 5, 6, and 7 of Figure 9 are formed in a Z-shaped path, a vertical path and a contour-filling path respectively. For surface 1, the three samples all look smooth with little side spatters. For surface 2, sample 5 appears to be regularly corrugated and rough. Sample 6 appears slightly rippled, but the accuracy is not high. Sample 7 has no apparent corrugations in the longitudinal direction. There are only slight traces of overlapping layers. For surface 3, all the samples look smooth, but the main body of the samples is higher than the edge of the samples.

#### 3.3.2. Macro Defect Mechanisms with Different Tool Paths

The defects in this set of experiments mainly occurred in the form of poor flatness and side spatters on surface 2. The side spatters are the result of the lateral wire feeding which was discussed in Section 3.1. Different layer-to-layer overlapping methods lead to great differences in the appearance of the flatness.

**Figure 10:**
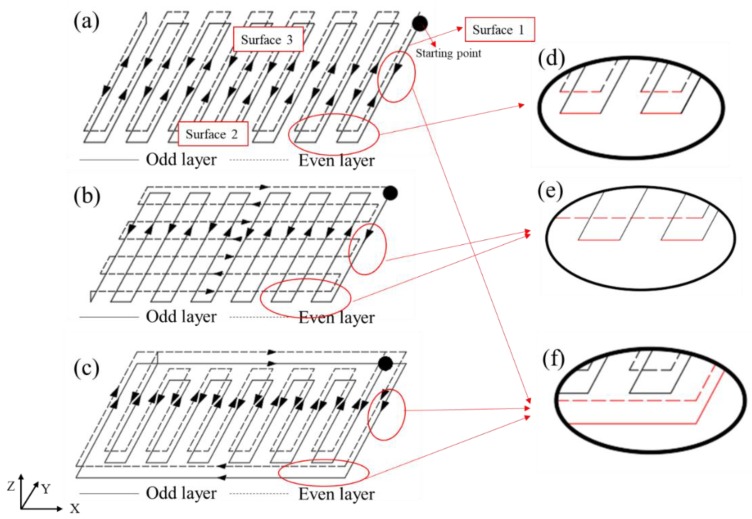
Schematic of (**a**) Z-shaped path; (**b**) vertical path; and (**c**) contour filling path. (**d**–**f**) mean three kinds of layer-to-layer overlapping methods.

**Figure 11:**
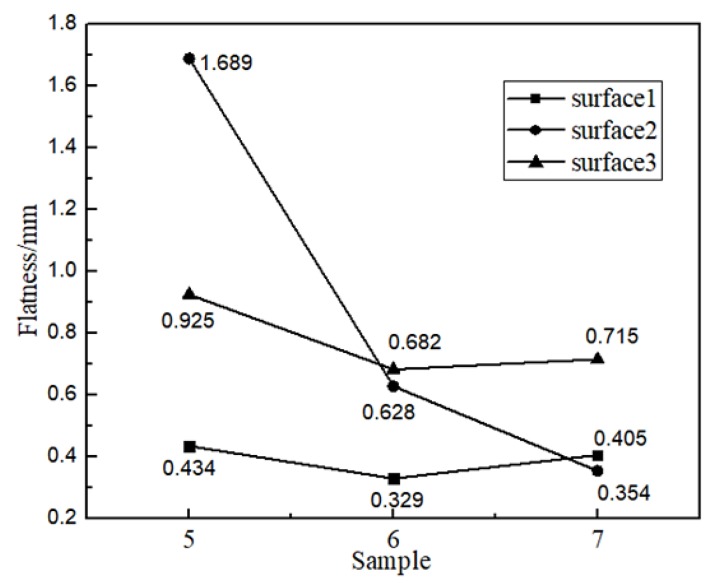
Flatness values of surfaces 1, 2, and 3 from samples 5, 6, and 7.

From the path schematic (Figure 10a–c), there are mainly three types of layer-to-layer overlapping (Figure 10d–f). The trajectory of each layer in Figure 10a is repetitive. The paths of odd layers are perpendicular to those of the even layers in Figure 10b. The paths in Figure 10c are frame with zigzag filled, that is, the frame is fabricated for every layer at first, then the zigzag paths are used to fill the frame. Three overlapping types were analyzed combined with the value of the flatness as follows:

1. The first overlapping type is shown in Figure 10d. The surface is composed of the metal that is located on the turning of each weld bead. The movement of the machine tool is continuous, and the weld bead has a certain width. Therefore, the path in the 180° corner of each layer is always in the form of a circle, and because the paths of each layer are repetitive, surface 2 becomes obviously corrugated during the fabricating process.
  Figure 11 reflects the flatness values of surface 1, surface 2, and surface 3 of the three paths. Surface 2 of sample 5 in Figure 9 is used with this overlapping method with a flatness value of 1.689 mm.
  The second overlapping type is shown in Figure 10e. The metal of odd layers is at the corners of each weld bead, and the metal of even layers is covered by a straight weld bead directly. In this way, the overlapping gaps produced by the odd layers are supplemented by the metal of even layers, and the even-numbered layers of molten metal complement the voids from the odd-numbered layers, reducing the amount of ripple phenomenon of surface 2.
2. The overlap patterns of surface 1 and surface 2 of sample 6 in Figure 9 are the same, but the values are 0.329 and 0.628 mm, a difference of nearly a factor of 2. This difference occurs mainly because the weld bead of surface 1 is much shorter than that of surface 2. The heat input is relatively concentrated, causing the even layer to fill the odd layers with more metal, thus reducing the flatness value. Furthermore, the accumulation of heat input tends to cause collapse on surface 1, which corresponds to the previous result.
  The third overlapping type is shown in Figure 10f. Each layer is a complete straight weld bead without turning of the path, so the surface can be smoother. There are only small scale patterns in the horizontal direction, which is the effect of overlapping of welding pool, an inherent phenomenon in wire arc additive manufacturing.
3. Surface 1 of sample 5 and surfaces 1 and 2 of sample 7 of Figure 9 are of the same overlapping type, according to the above description. Their flatness values are 0.434, 0.405, and 0.354 mm, which are almost the same and confirms the above-mentioned overlap mechanism.

In conclusion, under the three paths, the flatnesses of the surfaces 3 and 1 are almost the same, but the flatnesses of surface 2 of samples 5, 6, and 7 in Figure 9 are successively decreased from 1.689 mm to 0.628 mm to 0.354 mm. Therefore, different paths still have obvious regulation effects on the flatness defect of the surface. Choosing a reasonable path for parts helps to improve surface accuracy, reduce the amount of subsequent processing and improve the efficiency of wire arc additive manufacturing.

### 3.4. Effect of Wire Curvature on Macro Defects

#### 3.4.1. The Form of Defects

**Figure 12:**
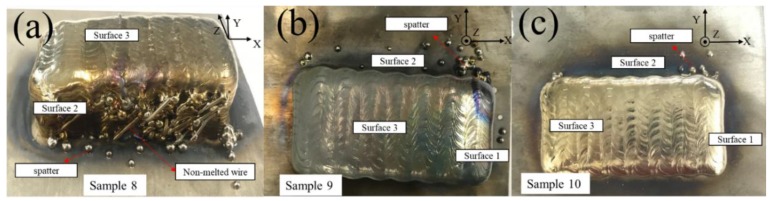
Macro images of samples 8, 9, and 10 with different wire curvatures: (**a**) 1/150; (**b**) 1/300; and (**c**) 0.

Figure 12 shows the defects existing in three samples with different wire curvatures. It can be seen clearly that the defects are mainly in the form of spatters and unmelted state. Samples 8 and 9 are fabricated using wires with curvatures of 1/150 and 1/300, and sample 10 is fabricated using a wire with a curvature of nearly zero after straightening, which can be regarded as a straight line. Surface 2 of sample 8 in Figure 12 shows that a large amount of wire poked out from the side of the sample in an unmelted state, and there are also droplets hanging onto surface 2 or spilling out. Surface 2 of sample 9 shows only a small amount of spatter. Surface 2 of sample 10 is free from spatters, and only droplets are present at the intersection of surface 2 and surface 1.

#### 3.4.2. Macro Defect Mechanisms with Different Wire Curvatures

**Figure 13:**
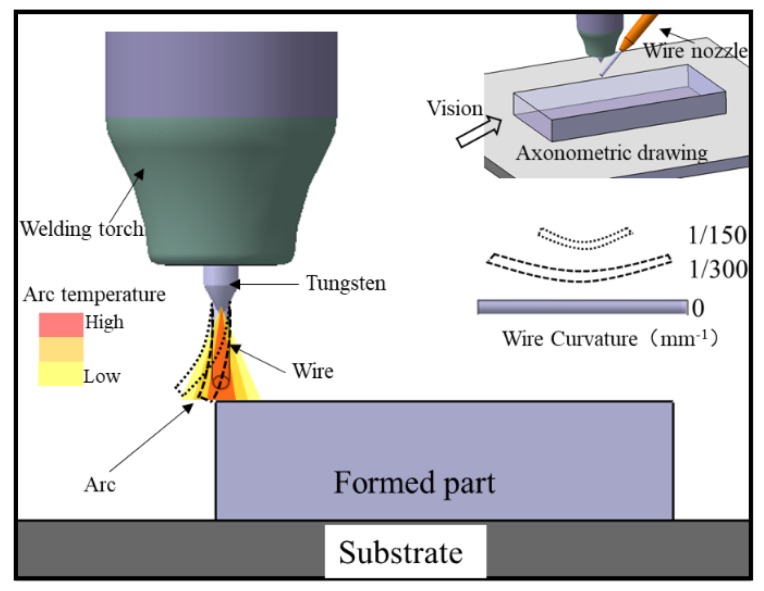
Schematic of the wire at the edge of the part for different curvatures.

The defects of this experimental set are mainly in the form of unmelted wire and side spatters on surface 2. The wire used in the fabricating process was provided by the wire manufacturer, and different heat treatment processes resulted in different curvatures when the wire was wrapped around the wire reel. As shown in Figure 13, the larger the curvature of the wire, the more bent is the extended wire. This problem is not noticeable when fabricating a part with a few layers. When fabricating a block whose height is much higher than the substrate, wire with different curvatures can bring different defects once the path is scanned to the edge.

As Figure 13 shows, when the wire is straight (with a curvature of 0), the front of the wire is located on the center of the arc where the heat input is the highest and the droplets can fall accurately on the trajectory of the movement. Almost no spatter appears. Spatter from sample 10 occurs because the position of the spatter is the starting point of each layer where the welding torch is lifted suddenly, making the AL longer at that moment and causing the spatters.

When the curvature of the wire is 1/300, droplets do not form at the center of the arc under the tungsten. The droplets are easily blown out of the sample under the arc force in a fastigiated direction. The spatters of sample 9 are caused by this.

When the wire is excessively bent with a curvature of 1/150, the front of the wire stays away from the concentrated area of the heat input, causing the heat input to be too low so that the wire cannot be melted sufficiently. As a result, the spatters appear at surface 2, and the wire cannot be melted and poked out from surface 2. Therefore, the poor melting of the wire makes the wire adhere to the formed surface easily. It also has a certain influence on the wire feeding system and it can impede the stability of the equipment.

The three samples of Figure 12 show three kinds of wires whose curvatures are 1/300, 1/150, and 0. It can be clearly seen that the unmelted wire of surface 2 is gradually reduced, and the the number of spatters likewise, which confirms that the wire produced by different processes has a significant effect on the spatters. The wire used in sample 10 is based on the selection of a lower curvature wire, and a straightener is applied, so the wire from the wire nozzle is nearly straight. Therefore, choosing a suitable wire curvature also provides an effective method of controlling the generation of macro defects such as spatters, which is beneficial to reducing defects in the block parts.

## 4. Conclusions

In this paper, four variables were selected to find the defect forms of the sample fabricated by wire arc additive manufacturing. The effects of heat input, layer thickness, tool path, and wire curvature on macro defects were investigated to establish the mechanisms of macro defects. The controlling methods also could be obtained by adjusting these parameters. According to the results and discussions, the main conclusions of this paper are as follows:

1. Improper heat input brings effect of side spatters, collapse, and unmelted wire. When the heat input is decreased from 261 to 173 J/mm, the size and number of spatters are reduced obviously and less degree of collapse is observed as well. However, unmelted wire occurs as the heat input is decreased. Even if decreasing the heat input can reduce defects, the main reason for the side spatters is the lateral wire feeding mode.
2. Improper layer thickness brings effects of side spatters and collapse. Different layer thicknesses affect the arc length, which can determine the metal transfer mode: no-droplet mode, tangent-droplet mode, or no-contact mode. Based on lateral wire feeding, the no-contact mode easily results in side spatters and the no-droplet mode easily results in collapse. Choosing a changeable layer thickness can effectively reduce those defects.
3. Different tool paths mainly lead to different surface flatness levels. Three overlapping types at the edge of the sample are proposed. It is meaningful to choose the appropriate tool path to fabricate the components in WFAM, which can efficiently reduce post-processing time and improve manufacturing efficiency.
4. Different wire curvatures bring side spatters and unmelted wire. In this paper, three kinds of wire with curvatures of 0, 1/300, and 1/150 were investigated. It was found that the more bent the wire, the more frequent are the spatters and the appearance of unmelted wire. Therefore, the development of wire standards or the use of a straightener in the manufacturing process can effectively improve the straightness of the wire and the reliability of manufacturing, which is of significance to the development of WFAM.

In summary, the most serious defects in WFAM are side spatters and collapse. Combined with the above results, it can be concluded that the main reason for these defects is that the wire is not melted in the arc accurately with improper variables. This paper illustrates the mechanism and proposes controlling methods so that these defects can be effectively reduced by choosing the appropriate heat input, layer thickness, and wire curvature, which is meaningful for the further application of WFAM.

## Figures and Tables

**Table 1:** Deposition parameters used in this research.

Sample	1	2	3	4	5	6	7	8	9	10
Experimental group	Group 1				Group 3					
			Group 2					Group 4		
Heat input (J/mm)	262	173	217	217	217	217	217	217	217	217
Layer thickness (mm)	1.05	1.05	1.05	1.1	1.05/1.1	1.05/1.1	1.05/1.1	1.05/1.1	1.05/1.1	1.05/1.1
Path	Z	Z	Z	Z	Z	Vertical	Contour filling	Z	Z	Z
Wire curvature (mm^−1^)	0	0	0	0	0	0	0	1/150	1/300	0
